# Birth weight and delivery practice in a Vietnamese rural district during 12 year of rapid economic development

**DOI:** 10.1186/1471-2393-13-41

**Published:** 2013-02-19

**Authors:** Huong Thu Nguyen, Bo Eriksson, Toan Khanh Tran, Chuc Thi Kim Nguyen, Henry Ascher

**Affiliations:** 1 Research Institute for Child Health, National Hospital of Pediatrics, 18/879 La Thanh road, Hanoi, Dong Da district, Vietnam; 2 Nordic School of Public Health, PO Box 12133, Gothenburg SE-402 42, Sweden; 3 Family Medicine Department, Hanoi Medical University, No.1 Ton That Tung Street, Hanoi, Vietnam; 4 Department of Social Medicine and Public Health, Sahlgrenska Academy, University of Gothenburg, PO Box 440, Gothenburg SE-405 30, Sweden

**Keywords:** Birth weight, Social and economic development, Sex ratio at birth, Rural Vietnam

## Abstract

**Background:**

Since the Doi Moi reform 1986 economic conditions in Vietnam have changed significantly and positive health and health care developments have been observed. International experience shows that improved economic conditions in a country can reduce the risk of perinatal mortality, decrease the risk of low birth weight and increase the mean birth weight in newborns. The Health and Demographic Surveillance Site (HDSS) FilaBavi in Bavi district outside Hanoi city has been operational since 1999. An open cohort of more than 12,000 households (52,000 persons) has been followed primarily with respect to demography, economy and education. The aim of this research is to study trends in birth weight as well as birth and delivery practices over the time period 1999–2010 in FilaBavi in relation to the social and economic development.

**Methods:**

Information about birth weight, sex, place and method of delivery, mother’s age and education as well as household economy of 10,114 children, born from 1999 to 2010, was obtained from the routine data collection in the HDSS.

**Results:**

Over the study period the mean birth weight remained at the same level, about 3,100 g, in spite of increased economic resources and technology development. At the individual child level we found associations between birth weight and household economy as well as the education of the mother. Hospital delivery increased from about 35% to 65% and the use of Caesarian section increased from 2.6% to 10.1%.

**Conclusion:**

During the twelve years studied, household income as well as the use of modern technology increased rapidly. In spite of that, the mean and variation of birth weight did not change systematically. It is suggested that increasing gaps in economic conditions and misallocation of resources, possibly to overuse of technology, are partly responsible.

## Background

During the last few decades, there has been a dramatic change in the economic conditions in Vietnam. Yearly income per capita has increased from $130 in the early 1990s to $900 in 2008. Poverty according to the World Bank definition (less than 1.25 US$ per person and day) has been reduced from 58% of the population in 1993 to 13% in 2008 [1]. The total health expenditure in Vietnam increased from 4.9% of GDP in 1999 to 5.9% in 2005. Expenditures for public health care and services decreased while expenditures for care by private providers increased [2]. Some health care and health effects have been noted. The percentages of women, who used at least one antenatal care visit, increased from 71% in 1997 [3] to over 80% in 2005 [4]. The proportion of low birth weight infants decreased from 9% in 2000 [5] to 7% in 2007 [6]. The infant mortality rate (IMR) decreased from 39 to 18 per 1,000 live born births and the maternal mortality ratio (MMR) decreased from 92 to 80 per 100,000 live births between 1997 and 2005 [4].

The Health and Demographic Surveillance Site (HDSS) FilaBavi in the rural district Bavi started in 1999. An open cohort of about 12,000 households has since then been followed regularly. From 1999 to 2009, the median self-reported yearly income per household in FilaBavi increased from 5,990,000 Vietnamese dong (VND) (18,932 VND = 1 US$ in 2010) to 15,000,000 VND, taking inflation, (118% according to International Monetary Fund) over the period, into account.

In a situation of economic growth but with possibly widening gaps in socioeconomic conditions within populations there is an urgent need for surveillance of health and well-being. A vulnerable group is children, particularly the newborn and the very young. Birth weight is an indicator of the mother’s health and nutritional status. It is also an indicator of a newborn’s chances for survival, growth, long-term health and psychosocial development [7]. Low birth weight (LBW, birth weight less than 2,500 grams) raises the risk of infant death and adverse health outcomes later in life such as asthma, coronary heart diseases, type 2 diabetes and hypertension [8-10]. LBW is a public health problem in most developing countries, where an estimated 15 per cent of births result in LBW babies. Reducing the incidence of LBW is one of the major goals in ‘A World Fit for Children,’ the Declaration and Plan of Action adopted at the United Nations General Assembly Special Session on Children in 2002 [5].

Several genetic and environmental factors influence the intrauterine growth of a fetus and hence the birth weight. The different strengths and prevalences of those factors lead to differences in birth weight between countries, ethnic groups and different social and economic contexts. At the household and individual levels variations occur due to a manifold of influences. For example demographic, social and economic conditions are known as determinants of health in general, including birth weight. It is so far not known if the economic changes in Vietnam have been associated with birth weight changes and changes in the proportion of low birth newborn. Therefore the primary aim of this paper is to study trends in birth weight together with the proportion of LBW newborn over the time period 1999–2010 in FilaBavi. There is also an aim to study trends in delivery place, presence of medical attendance and delivery type, particularly the use of Cesarean section (CS). Finally we want to study how birth weight and delivery characteristics are associated with economic and educational characteristics of the mother and the household. This will be done at the macro level, over time, as well as at the level of individual households.

## Methods

### FilaBavi

The HDSS FilaBavi started with a baseline study in 1999. Over 12,000 randomly selected households, with just over 50 000 persons, in 69 clusters (hamlets) in Bavi district 60 km west of Hanoi were included and interviews were conducted to obtain information about the households and the individuals living in these. At the same time the demographic monitoring started with household visits every three months to record vital events, births, migration and deaths. The site has been running since, with quarterly follow-up interviews. Renewed household surveys have been undertaken in 2001, 2003, 2005, 2007 and 2009 [11].

The household surveys have given information about household economy and living conditions as well as individual information like education and occupation. The quarterly visits have been used to obtain information about vital events. The total number of live born children reported from 1st January 1999 to 31st December 2010 was 10,114. This paper presents results concerning these children.

### Information and variables

Information on birth weight, sex of the child, state of twins and date of last menstruation before pregnancy were reported by the mothers. The date for last menstruation was intended to be used for the estimation of the gestational age at birth. This information however, must be considered as fairly imprecise. The difference between the date of birth and the date for the last menstruation as reported by the mother can however be assumed to be rather strongly correlated to the gestational age at birth and is used as a proxy for the gestational age, subsequently referred to as Gestational age proxy (Gap).

The following information about the newborns was used in the analysis of associations: birth weight, sex, Gap, place of delivery, medical attendance at delivery and type of delivery, specifically CS.

For the mothers we used age and education classified in three levels (primary, secondary and higher) from the household surveys. Farming was the most frequent occupation in the rural area. Occupation was strongly correlated to education and was therefore not used in the analysis. About 94% of the population belonged to the Kinh ethnic group. We don’t present details about the other ethnic and occupational groups because they are small and therefore not considered in the analysis.

Information describing economy was taken from the household surveys. The reported yearly household income and the household assets available (according to a specified list) were considered as indicators of economic resources. The number of household members was also used in the analysis.

### Statistical analysis

The main outcome variable is birth weight. We also give some results for the dichotomized, less informative but often used variable LBW. To explore possible associations with various background variables, linear regression models were used. As some mothers gave birth to more than one child during the 12 years, we used multiple two-level mixed linear models. The difference in the results between this approach and ordinary regression is indeed minor. LBW was analyzed using parallel multiple logistic models.

To study the development over time for birth weight and delivery characteristics we used simple linear regression of outcomes on the independent time variable. Two approaches for analysis of associations between outcomes and background variables were used. In one we used all data in one model. However, associations might change over time and year-wise analyses were also used to attempt identification of such tendencies. All computational work was carried out using STATA, version 11.

### Ethical considerations

This study was a part of larger collaborative project between Vietnamese and Swedish researchers. It was approved by Hanoi Medical University and Ministry of Health, Hanoi and Bavi district People Committees and Bavi District Health Center, Hanoi, Vietnam. All households approached were informed about the purpose of the studies and their right to decline participation or withdraw. It was also clarified that strict rules for confidentiality should be adhered to. Information was given that all results shall be continuously disseminated to communities and authorities as aggregated information. Less than 1% of the households approached refused participation.

## Results

### Children born 1999–2010

Out of the 10,114 children born in FilaBavi, 5,389 were boys and 4,725 girls. The number of mothers was 6,860, where 4,093 had one birth and 2,305 had two. The maximum number of reported births for one mother was five. Birth weight information could not be obtained for 98 children, less than 1%. Not all children born in 1999 were included in the study because the first household survey was completed only in April 1999. Eight children were excluded from the analysis due to generally poor information. Table 1 gives an overview of the children included in this study.

**Table 1:** Live born children by year and sex, FilaBavi 1999–2010

**Year**	**1999**	**2000**	**2001**	**2002**	**2003**	**2004**	**2005**	**2006**	**2007**	**2008**	**2009**	**2010**
All Live born Boys	306	417	390	422	466	460	481	479	486	474	545	464
All Live born Girls	295	401	370	393	419	410	370	377	434	433	431	391
All Live born	601	818	760	815	885	870	851	856	920	907	976	855
Reported Twins	1	2	2	5	17	12	13	8	12	12	11	10
Sex ratio at birth SRB*	1.04	1.04	1.05	1.07	1.11	1.12	1.30	1.27	1.12	1.09	1.26	1.19

* SRB larger than 1.21 are statistically significantly (p < 0.05) larger than the normal 1.05-1.08.

The absolute number of births increased over the period. Comparing 2000 and 2009 the increase was about 30% for boys and 10% for girls. As a consequence, the male to female sex ratio at birth (SRB) increased from 1.04 in 1999 to 1.19 in 2010 (Table 1). For some years it was larger than 1.21 which is the limit for being statistically significantly (at the 5% level) more than 1.04-1.06 which is mostly assumed to be normal [12].

### Birth weight and birth weight trends

The mean birth weight for all boys born 1999–2010 was 3,136 grams with a standard deviation of 451 grams. For girls the mean was 3,057 grams with a standard deviation of 421 grams. The WHO growth standards published in 2006 [13] reported the means for boys and girls as 3,346 and 3,232 grams respectively, that is a little more than 200 grams higher than the means found in this study.

Table 2 shows the trends of birth weight over time. There were no strong tendencies for a systematic change in birth weight over time for the main group, singleton children. The results of a simple regression of birth weight on birth year actually showed an average decrease of 3.4 grams per year and a p-value which was 0.01. The same analysis by sex gave a yearly decrease for boys of 4.5 grams and of 2.5.grams for girls. The former decrease was statistically significant, the latter not. The mean birth weight for twins remained at the same level (Table 2) and the estimates of LBW percentages which stayed at a level of about 5%.

**Table 2:** Estimates of mean birth weight (g), FilaBavi 1999-2010

**Year**	**1999**	**2000**	**2001**	**2002**	**2003**	**2004**	**2005**	**2006**	**2007**	**2008**	**2009**	**2010**
Birth weight boys *	3,145	3,178	3,152	3,127	3,189	3,158	3,158	3,130	3,120	3,146	3,103	3.144
Birth weight girls *	3,072	3,064	3,080	3,041	3,095	3,097	3,087	3,009	3,059	3,056	3,066	3029
Birth weight total*	3,109	3,121	3,117	3,085	3,144	3,130	3,127	3,077	3,091	3,102	3,087	3,091
Birth weight twins **	2,600	2,800	2,550	2,440	2,411	2,700	2,107	1,875	2,383	2,466	2,563	2,570
LBW boys %***	7.2	3.6	5.4	6.2	4.9	3.7	5.2	5.2	6.0	5.3	5.0	3.4
LBW girls %***	5.1	4.2	5.7	7.6	5.7	4.4	5.7	6.4	4.8	4.4	4.6	5.6
Birth weight children born in hospitals**	3,067	3,096	3,052	3,018	3,091	3,081	3,051	3,018	3,041	3,046	3,035	3,064
Birth weight children delivered using CS**	2,912	3,173	2,952	3,013	3,195	3,101	3,174	3,085	3,133	3,211	3,071	3,211

* Singleton.

** Both sexes.

*** Percentage of children with birth weight less than 2500 grams, all live born.

A detailed description of the birth weight distributions for boys and girls in 2010 is given in Table 3. The median and the quartile deviation are almost constant over the years studied. The range varies due to individual extreme values but the difference between the 1% and 99% percentile is again very stable for the 12 years.

**Table 3:** Central tendency and variation of birth weight for boys and girls, 2010

	**Mean, 95% confidence interval**	**Standard deviation**	**Median**	**Quartile deviation**	**Range**	**Percentile difference 99%-1**	Number of children*
Boys	3144g (3106;3181)	410g	3200g	500g	2900g	2200g	464
Girls	3029g (2990;3068)	391g	3000g	500g	2500g	2000g	391

*Information about sex is missing for two children. Boys and girls studied.

The birth weights of children delivered in hospital, and specifically for those delivered using CS, are also shown in Table 2. We found no systematic change in birth weight for twins and children delivered in hospitals. For CS delivered children, we found increasing mean birth weights (p < 0.01).

### Background variable development over time

The mean values of the Gap indicator did not change systematically over time. The percentage of mothers giving birth in hospital roughly doubled over the studied time period. The increase was numerically not as fast for girls as for boys but the difference between the increases was not statistically significant. Overall, delivery in hospital was more common for boys (p < 0.001). Also, the use of CS became more frequent both for boys (2.6% in 1999 and 12.7% in 2010) and girls (2.7% in 1999 and 7.4% in 2010). These differences are statistically significant (p < 0.05) (Table 4). The percentage of deliveries with skilled birth attendance was high regardless of the sex of the child (95.1% in 1999 and 98.4% in 2010).

**Table 4:** Delivery characteristics, FilaBavi 1999–2010

**Year**	**1999**	**2000**	**2001**	**2002**	**2003**	**2004**	**2005**	**2006**	**2007**	**2008**	**2009**	**2010**
Delivery in hospital boys %	36.3	38.4	42.4	48.9	50.3	49.7	50.8	58.0	61.6	64.0	67.25	68.5
Delivery in hospital girls %	34.9	35.5	40.9	40.3	45.1	45.0	48.4	53.6	54.4	62.8	61.2	61.6
Delivery attendant (%)*	95.1	96.9	97.5	97.6	98.9	98.9	99.2	99.2	99.7	99.9	99.8	98.4
CS boys %	2.6	1.7	2.3	5.0	5.6	6.7	7.7	7.5	11.6	11.2	11.1	12.7
CS girls %	2.7	2.0	2.7	3.8	4.1	4.0	4.9	6.9	6.9	10.6	12.8	7.4

* Both sexes. Overall attendance Boys 98.55%, Girls 98.44%.

The mean age of mothers did not change markedly over the period under study. The percentage of mothers with education higher than high school increased from 4.3% to 16.5% during the period while the percentage of mothers with less than secondary school education decreased from 82.7% in 1999 to 52% in 2010. The proportion of ethnic Kinh mothers remained stable at about 94% over the years.

The median reported yearly household income increased from 5,990,000 VND in 1999 to about 15 000,000 VND (adjusted for inflation) in 2009 (150% increase). For the households in the lowest wealth-index quintile starting at 2,600,000 VND the increase was to 4,390,000 VND (69% increase). The highest quintile starting at 10,800,000 increased by 227% to 35.400,000. Reported household health expenditures roughly doubled during the period and were not much different between the income quintiles except that the lowest income group also reported somewhat lower health expenditure. The number of household assets increased modestly from 1999 to 2010. Household size remained stable.

### Analysis of birth weight variation

Two variables, Gap and child sex, were statistically significantly correlated to birth weight in all yearly multiple regression analyses as expected. Gap showed the strongest correlation coefficient throughout, around 0.30 and the regression coefficient indicated about 33 grams weight increase per week increase of Gap. The child sex variable showed a significant boy girl difference somewhat less than 100 g (p < 0.001). Mother age (increasing age associated with increasing birth weight), number of household members (more members - lower birth weight) and household assets (more assets - higher birth weight) occurred as significant for several but not all years.

In the overall multiple regression analysis, birth weight was statistically significantly associated to Gap (35 g per week), child sex (106 g boy - girl difference), hospital delivery (106 g lower weight than delivery in commune health center (CHC), education (36 g per education level) and number of household assets (12 g per asset) (p < 0.01). The very modest negative trend of birth weight over the years was not statistically significant in this analysis.

In an analysis restricted to hospital delivered children we identified a statistically significant association between use of CS and birth weight. The mean weight for babies delivered with CS was 65 g higher (p < 0.01) than for other babies. The mean birth weight for children where the mother was not of Kinh ethnicity was similar to the mean birth weight of the majority population.

CS is by necessity associated with delivery in hospital. We therefore studied the associations between hospital delivery and background factors only for deliveries other than CS. Statistically significant associations were observed for child sex, mother’s age and education and economic status. Boys were more often delivered in hospitals (from 36.3% in 1999 to 68.5% in 2010) than girls (from 34.9% in 1999 to 61.6%). The mother’s age was not linearly associated; there is a peak in hospital delivery for the age group 20–29. Economic status in this analysis turned out to be best represented by the reported monetary income.

Increasing use of CS is monotonically associated primarily with increasing education and increasing mother age. There are tendencies for associations also with increasing economic status and male sex of the baby (p < 0.05).

No model for birth weight had an R^2^ larger than 0.15, which means that the variation in the independent variables explained 15% or less of the variation in the dependent variable. The corresponding values for low birth weight were even lower.

The reported household monetary income appeared to be statistically significant associated with birthe weight in few analyses where the household assets variable was included. These variables are fairly strongly correlated to each other (r = 0.6). If the assets variable is removed however, income replaces it as being significantly associated with birth weight.

## Discussion

The main finding in this study of the comparatively long time series is that the overall mean birth weight has been stable over the years in spite of a rather drastic economic and technological development. Most previous studies in Vietnam, and other countries, have found increases in the mean birth weight and reductions in the incidence of low birth weight over time periods with improved economy [10,14-16]. One study even showed that the low birth weight proportion decreased in spite of an increased rate of multiple births [17]. Some possible explanations have been mentioned. Improvement of socioeconomic conditions can lead to better nutrition of mothers during pregnancy and increased mother’s weight gain during pregnancy [10,14-16]. Increased economic resources can facilitate access to antenatal care. An interesting contradictory result is given in a study of term singleton births in the United States where the mean birth weight decreased from 1990 to 2005. The finding could not be explained by maternal and neonatal characteristics or by trends in induced labor or CS. Neither did the observed decline in gestational age at birth, which is normally strongly correlated to birth weight, explain the decreasing birth weight [18]. The present results show no systematic changes over time in the gestational age proxy indicator used.

In the yearly analyses at the level of individual children we find associations between birth weight and indicators of economic resources as well as education. This is not a contradiction to the finding that the mean birth weight did not increase over years. First a certain time lag from economic changes to birth weight effects should be expected. Further, the general economic improvement does not necessarily influence on the general birth weight level, particularly if the economic and nutritional inequalities remain or increase. Over the years there can also be changes in household priorities for the use of economic resources.

UNICEF has suggested that economic development alone does not automatically lead to improvement in nutrition. Good nutrition is an outcome of many factors both biological and social [19]. Most pregnant women in rural areas are not covered by any social insurance scheme [20]. The present study shows that household health expenditures roughly doubled during the period and were not much different between income quintiles. This means that poor households spend a much higher share of their total income on health than the higher income groups. Rural mothers have to pay themselves for antenatal care if they are not covered by any social insurance and even if they are covered, may still have to pay themselves for delivery or antenatal care in hospital, particularly if they come directly, without referral, to a hospital.

The present study shows that the percentages of women who delivered in hospitals as well as women who delivered with CS, increased during the time period. This tendency might be an indication of increased confidence in technology. Along the same line, the use of ultrasound has become increasingly common. Possible overuse of ultrasound has been seen in another study in Vietnam in 2007 [21].

Compared to results from urban Hanoi, the pregnant women in FilaBavi attended antenatal care later, had fewer visits and much fewer medical services [22]. The health expenditure of pregnant women may to a large extent be payment for health technology rather than for nutrition and good antenatal health care. E.g. the prevalence of anemia of pregnant women in Vietnam did not change noticeable between 2001 (32.2%) [23] and 2008 (31.4%) [24].

A finding is that the percentages of women, who gave birth in hospitals as well as delivered with trained attendance, increased over the twelve years. Vietnam has a strict two child policy. From a recent study in FilaBavi, we know that the proportion of delivery in hospitals is higher among women with higher education and among mothers without previous sons in FilaBavi [25]. Most likely all women want to have good delivery care for themselves and their newborns and believe that the quality of delivery care is better in the hospitals than in the commune health centers. There is no formal barrier for women to go directly to any hospital to give birth, if they can pay for themselves. According to the Health Strategy and Policy Institute, about 90-95% of all patients in the Central Obstetric Hospital in Hanoi, including mothers with low economic condition, went directly to the hospital without transferring from local hospital [26]. The main reason for this care-seeking behavior can be that people have higher confidence in the professional staff, medical equipment, and infrastructure of the central hospitals [26].

We found that the use of CS increased over the 12 years in FilaBavi along with the increase of economic resources and mother’s education. This is in accordance with the result from a recent study in FilaBavi, in which CS use increased with education level and among women living at lowland areas or in richer households [25]. CS has increased also in other countries. The highest percentages of CS in Asia were in China (46.2%). The second country was Vietnam (35.6% in 2007 in three obstetric hospitals) [27]. A reason for CS without medical indication can be that families want to choose a good time and day for operation, that the operation is believed to be safe and that the child will have a good future life as a result. There are beliefs that CS will prevent asphyxia for newborns and that it is a safe and painless technique for both mother and baby. A study in nine Asian countries showed that CS is associated with improved perinatal outcome, but it also pointed to increased risk of neonatal intensive care treatment, maternal death and maternal morbidity [27]. The rate of CS has increased in FilaBavi since 1999 to 2010 but we do not have information about the distribution of medical and non-medical indications.

During the last 12 years, the male to female sex ratio at birth (SRB) has increased in Filabavi. The sex ratio at birth in 2010 was quite far from the normal. In Vietnam as a whole, SRB increased from 1.05 in 2001 to 1.12 in 2006 [28]. This is similar to results from other studies in Vietnam [28] and other Asian countries, particularly India and China. It has been estimated that around 80 million females in China and India are “missing” [29]. Sons are considered more valuable to parents than daughters. The first son will maintain the family line and be responsible for carrying out the cult of the ancestors. Almost all parents want to give their house and other wealth only to their sons, especially the first son [30]. Another reason for the high SRB is that stricter population regulations were also introduced in Vietnam at the end of 1980s saying that families should not have more than two children [28]. Parents therefore may try harder to have at least one son. It is not difficult for women to know the sex of the foetus very early through ultrasound scanning and women can decide freely about early abortion. One can suspect that the beliefs and traditions behind the elevated SRB also influence the choices of delivery place and method as mentioned above.

This study gives a picture of birth weight trends in FilaBavi during twelve years from 1999 and the incidence of LBW, delivery place, presence of medical attendance at delivery and type of delivery, particularly the use CS. We also found an association between birth weight and delivery characteristics on the one hand and economic condition of the household as well as educational characteristics of the mother on the other.

The births studied are assumed to be all or close to all live births that actually took place in FilaBavi 1999–2010. Different small studies have been done comparing the numbers of reported births and deaths in FilaBavi with the official information given by local authorities. The latter reports roughly 10% fewer births. The deficit is higher regarding deaths [31].

The quality of the birth weight information used must be judged knowing how it was obtained. Mothers have reported the information they received in hospitals or community health centers immediately after delivery. In the Commune Health Centers and the District hospital, the newborn children were weighed and measured directly after delivery wearing light clothes. The birth weight, recorded in the clinical records of the district hospital or in the delivery notes of the commune health station, was adjusted for the weight of clothes.

In Vietnam, it is considered important to know and remember the birth weight of children. Three months after delivery, the field workers interviewed mother about birth weight, why recall bias should be low. We have also compared information of birth weight between the different kinds of interview, quarterly interviews in FilaBavi 1999–2010 and the quarterly interviews in FilaBavi and DodaLab 2008–2010 [25]. The results are that the estimated means of birth weight match very well for both sexes.

Birth weight was measured by different health staffs working differently and using different equipment. Poor standardization can create bias and excess random variation. This source of error is shared with many studies and difficult to eliminate in any context. The health staff may also report the birth weight to the mother incorrectly, e.g. with a tendency to “increase” for low weight babies and “decrease” for high weight babies as a wish to avoid extremes and thereby “please” the mother. This could lead to underestimation of the proportions of low birth weight children and to a biased estimate of the standard deviation (underestimated). Gifts to the health care staff, often monetary, are important parts of the Vietnamese health care system.

To explore possible bias a small study was done. For each year, 1999–2010, we collected the birth weight information for 30 randomly selected children from the records in the Bavi district hospital and compared the estimated means of birth weights with those obtained from the interviews with mothers. There were no trends over the 12 years in the birth weight means estimated using this particular data. The overall means were 3,110 g for boys and 3,047 g for girls. The corresponding estimates using the mother reports were 3,136 and 3,057 g, i.e. differences are 26 and 10 g. We also collected birth weight data for 40 children in each of two health stations for two years, 2005 and 2009. There was no large difference between the health station and the hospital data.

## Conclusion

During the studied twelve years there were improvement of economic conditions and education as well as increased expenditure for health care and use of health technology in a rural area of Vietnam. Surprisingly, no positive secular trend in birth weight was observed in this period. Some reasons can be that the increased economic resources are not distributed in an equitable way or not used in the best possible way. The research was carried out in one rural area of Vietnam and the results may not be representative for other rural areas of Vietnam. To better understand the mechanisms behind this result, similar studies in other areas of Vietnam would be needed. In future studies birth weight information should ideally be obtained from validated delivery registers to avoid biases.

## Competing interests

The authors declare that our findings have not been influenced by our personal or financial relationships with other persons or other organizations.

## Authors’ contributions

HNT led and supervised the fieldwork and data management. She also drafted and completed this paper. BE assisted in the research design as well as in the statistical analyses, interpretation of results and revising the manuscript. HA, TTK and CNTK were involved in the design of the study, supervised the study and revised the manuscript. All authors have read and approved the final manuscript.

## Pre-publication history

The pre-publication history for this paper can be accessed here:

http://www.biomedcentral.com/1471-2393/13/41/prepub
